# Safety of Additional Local Anesthetics Soon After Liposomal Bupivacaine: A Retrospective Cohort Study

**DOI:** 10.7759/cureus.112872

**Published:** 2026-07-17

**Authors:** Daniella Veloria, Lei Xu, Elizabeth Rader, David Ha, Daniel Gessner

**Affiliations:** 1 Department of Pharmacy, Stanford Health Care, Palo Alto, USA; 2 Department of Anesthesiology, Perioperative and Pain Medicine, Stanford University, Palo Alto, USA; 3 Department of Quality, Stanford Health Care, Palo Alto, USA

**Keywords:** liposomal bupivacaine, local anesthesia systemic toxicity, local anesthetic, peripheral nerve block, regional anesthesia and chronic pain

## Abstract

Introduction

Liposomal bupivacaine (LB) produces a prolonged serum bupivacaine level. It carries a warning to avoid additional local anesthetics for 96 hours due to a theoretical additive risk of local anesthetic systemic toxicity (LAST). Whether patients exposed to additional local anesthetics soon after LB have a clinically detectable increased risk of LAST is unknown.

Materials and methods

Data from a single academic center were used in a cohort study of adults who received LB intraoperatively. The exposure was to additional local anesthetics within 96 hours of LB administration, and the outcome was a composite of electronic medical record data indicating possible LAST. The cumulative incidence and hazard of possible LAST (with weighted adjustment for propensity to exposure to additional local anesthetics) were calculated. A propensity score-matched sensitivity analysis was performed using an outcome based on a manual chart review.

Results

We identified 4306 patients who received LB, of whom 466 (12.1%) were exposed to additional local anesthetics within 96 hours of LB. There was no significant difference in the cumulative incidence of possible LAST between exposed and unexposed patients (0.9% vs. 1.5%, p = 0.28) as compared to unexposed and no significant increase in the unadjusted hazard (HR 0.51, 95% CI 0.18-1.42, p = 0.21) or the adjusted hazard (HR 0.25, 95% CI 0.07-0.89, p = 0.033) of possible LAST after exposure to additional local anesthetic. The sensitivity analysis had similar findings.

Conclusions

We found no evidence that exposure to additional local anesthetics within 96 hours of LB administration increases the hazard of possible LAST.

## Introduction

Liposomal bupivacaine (LB) is a liposome-encapsulated local anesthetic indicated for single-dose perioperative administration and intended to produce a prolonged local anesthetic effect [1]. Infiltration of LB at a surgical site results in a serum bupivacaine level that has a bimodal distribution: an early peak soon after administration, followed by a later peak between 10 and 36 hours post-administration, and a prolonged half-life, with detectable levels lasting 96 hours or more [2]. As a result of this pharmacologic profile, there is a risk for delayed-onset local anesthetic systemic toxicity (LAST) associated with LB [3].

The original October 2011 United States Food and Drug Administration (FDA)-approved label for LB acknowledged that significant drug levels could persist for 96 hours and warned against the administration of other forms of bupivacaine during that period [4]. After a 2018 FDA committee meeting [5] in which concerns were raised about using non-bupivacaine local anesthetics soon after LB, the FDA labeling was expanded to advise against any additional use of local anesthetics for 96 hours [6]. The 2020 European Medicines Agency Summary of Product Characteristics also advises caution with using additional local anesthetics after LB because of an additive risk of systemic toxicity [7].

While the use of LB alone, like all local anesthetics, is associated with some risk of LAST [8,9], a clinically relevant increase in LAST risk from additional local anesthetic use soon after LB has not been well characterized. A prolonged prohibition on additional local anesthetic administration after LB is difficult to enforce, may limit opportunities to use LB, and can constrain treatment options for patients who require subsequent procedures soon after receiving LB. Our primary objective was therefore to estimate whether there is a clinically detectable hazard of increased toxicity associated with exposure to additional local anesthetics within 96 hours of LB administration. We also investigated whether our hazard estimate was sensitive to the method we used to determine toxicity.

## Materials and methods

This was a retrospective observational cohort study of adult patients who received LB at a large academic medical center. Our Institutional Review Board approved this study with a waiver of informed consent. The STROBE statement guides the structure of this manuscript [10]. Our study population comprised adult patients who received LB; our exposure was additional local anesthetics administered within 96 hours of LB administration; and our outcome was a composite indicator of possible LAST.

Patient selection and data collection

From our electronic medical record (EMR), we extracted all administrations of LB to patients aged 18 or older from October 30, 2014 (the date that LB was first administered at our institution) to February 9, 2022 (the date when our hospital first implemented comprehensive policies and practices to prevent exposure to additional local anesthetics during the 96 hours after LB administration). In cases where patients had been exposed to LB more than once, we included only the initial dose of LB in the analysis. We extracted all other data from our research data warehouse (RDW) [11]. Manual chart review for the sensitivity analysis was performed using standard clinician access to our EMR.

Predictors and potential confounders

Based on clinical judgment, we identified demographic and clinical factors that may predict exposure to additional local anesthetics within 96 hours after LB. This included age, legal sex (the only available sex/gender field in our RDW), language proficiency, Black, Indigenous, or Person of Color (BIPOC) status, weight, body mass index (BMI), procedures occurring on the date of LB administration and up to four days later, comorbidities, history of chronic pain or opioid use disorder, and length of stay.

For procedures, we used the NBER ICD-10 to ICD-9 crosswalk [12], as well as the HCUP Surgery Flags Software [13] data (v2015.1 for ICD-9 and v2022.1 for CPT; Agency for Healthcare Research and Quality (AHRQ), Rockville, MD, USA) to identify both narrowly defined (“narrow,” usually major therapeutic procedures) and broadly defined surgeries (“broad,” major diagnostic and invasive minor procedures). We considered all “narrow” procedure codes on the day of LB administration to represent the index surgery. We used HCUP Clinical Classification Software [14] data (v2015.1 for ICD-9 and v2022.1 for CPT; Agency for Healthcare Research and Quality (AHRQ), Rockville, MD, USA) to classify index surgeries by organ system group, allowing multiple group assignments for complex multi-system index surgeries [15]. We excluded patients without an identifiable index surgery.

For diagnoses, we collected all diagnosis codes for all patients through postoperative day (POD) 7, counting from the index surgery, and used the NBER ICD-9 to ICD-10 crosswalk [12] as necessary. We used the R package comorbidity (v1.0.5; R Foundation for Statistical Computing, Vienna, Austria) to calculate van Walraven (VW) Elixhauser-based [16] comorbidity scores for each patient, using all diagnosis codes through the day of index surgery. We also used ICD-10-CM codes identified by Samples et al. to determine whether patients had a history of chronic pain or opioid use disorder [17]. We excluded patients without diagnosis codes in our RDW.

Exposure assessment

We extracted medication administration data from the RDW to determine whether a patient was exposed to additional local anesthetics soon after LB. From one to 96 hours after LB administration, we identified the first exposure to bolus or continuous local anesthetic. We ignored all local anesthetic exposures we deemed, based on clinical judgment, to be low risk; this excluded all cutaneous, mucosal, and oral routes of administration, as well as non-intravenous injections of lidocaine of less than or equal to 5 mL at a concentration of 2% or less. We excluded patients with no MAR data.

Outcome assessment

Our primary outcome was a composite of possible LAST, consisting of death, administration of lipid emulsion, or diagnosis codes indicating LAST or signs or symptoms of LAST. We excluded lipid emulsion doses administered within one hour of other forms of parenteral nutrition. Codes for possible LAST have been used by other investigators assessing the incidence of LAST and included diagnosis codes for ventricular fibrillation/tachycardia, unspecified convulsions, cardiac arrest with unspecified cause, and cardiac complications resulting from a procedure, as well as procedure codes for cardiopulmonary resuscitation [18,19]. For each patient, we identified the first occurrence of the composite outcome, from the day of index surgery through hospital discharge or POD 7, whichever came first.

Inverse probability of treatment weighting

Including all patients with complete data, we used inverse probability of treatment weighting to adjust for factors that might differ between the exposed and unexposed cohorts. Using the R package WeightIt (v1.3.2; R Foundation for Statistical Computing, Vienna, Austria), we calculated balancing weights based on a covariate-balancing propensity score that estimated the average treatment effect on the treated. The propensity score included predictors that we believed would influence a patient’s propensity for exposure to additional local anesthetics: age, weight, BMI, sex, BIPOC status, English language proficiency, preoperative and postoperative length of stay, VW Elixhauser comorbidity score, prior diagnosis of chronic pain or opioid use disorder, index surgery category, and exposure to additional procedures on POD 1 through 4.

Statistical analysis

Because we used a convenience sample of all eligible patients at our institution, we did not perform a power calculation. We assumed the missingness was random and excluded patients with any missing data. We assessed the balancing performance of our inverse probability of treatment weighting using absolute standardized differences (ASD) calculated with the R package cobalt (v4.5.5; R Foundation for Statistical Computing, Vienna, Austria), with an ASD <0.10 considered adequate balance. We used the chi-squared test to compare cumulative frequencies between cohorts and the R package survival (v3.7; R Foundation for Statistical Computing, Vienna, Austria) to generate Kaplan-Meier curves and calculate Cox regression-based unweighted and weighted hazard ratios.

Sensitivity analysis

We built a pair of matched cohorts to investigate whether outcome assessment via manual chart review would be more sensitive in detecting possible LAST. We selected a subset of patients from our primary analysis and used the same exposure assessment criteria. We included patients who underwent one of the two most common index surgery categories (orthopedic or gastrointestinal/abdominal surgery) and who were admitted for at least 72 hours after their index surgery, and excluded patients who were admitted before their index surgery, who were first exposed to higher-risk additional local anesthetic more than 36 hours after their index surgery, or who had any subsequent procedures through POD 4. Using the same predictors as for our inverse probability of treatment weighting, we used the R package MatchIt (v4.5.0; R Foundation for Statistical Computing, Vienna, Austria) to calculate a new logistic regression-based propensity score and perform 1:1 nearest neighbor matching with a caliper of 0.2 to create matched cohorts of exposed and unexposed patients. We assessed balance using matched variables in ASD, with an ASD <0.10 indicating adequate balance.

For our chart review outcome, we assembled a comprehensive list of signs and symptoms of potential LAST based on prior studies that used clinical documentation and FDA reports to assess the risk of LAST after LB administration [8,9,20]. This included 19 signs and symptoms of serious, neurologic, and/or cardiac complications (Table 1). One investigator (DV) blinded to exposure status then reviewed the charts of all members of the matched sensitivity analysis cohorts, using a predefined strategy (Table 2) and recording data in a REDCap database [21,22]. The strategy searched for any signs and symptoms occurring from the instant of LB administration during the index surgery through hospital discharge or POD 4, whichever came first.

**Table 1:** Signs and symptoms of possible LAST used for sensitivity analysis ICU: intensive care unit, LAST: local anesthetic systemic toxicity

Category	Sign/symptom
Serious	Code
	Death
	ICU admission
Neurological	Altered mental status
	Blurry vision
	Hypoventilation
	Dizziness
	Dysarthria
	Metallic taste
	Loss of consciousness
	Severe/persistent nausea
	Perioral numbness
	Seizure
	Tinnitus
Cardiac	Bradycardia requiring treatment
	Hypotension
	Malignant ventricular arrhythmia
	New atrial arrhythmia
	Tachycardia requiring treatment

**Table 2:** Predefined search strategy for sensitivity analysis

Sign/symptom	Criteria
Altered mental status	Chart search “confusion” or “delirium” or “unarousable”, determine if patient experienced or documented. Open nursing flowsheet “Delirium”, determine if patient experienced or documented.
Bradycardia requiring treatment	Open medications list, filter for “glycopyrrolate”, “atropine”, or “epinephrine”, determine if administered. Chart search “pacing”, determine whether transcutaneous or transvenous pacing is documented.
Blurry vision	Chart search “blurry vision”, determine if patient experienced or documented.
Code	Chart search “code”, determine whether code called or documented.
Dizziness	Chart search “dizziness”, “vertigo”, “lightheadedness”, “unsteadiness”, determine if patient experienced or documented.
Dysarthria	Chart search “dysarthria” or “slurred speech”, determine if patient experienced or documented. Open nursing flowsheet “Language/Speech”, check if “slurred” is documented.
Death	Open “Discharge Summary” note, determine if death date/time is documented.
Hypotension	Open ICU nursing flowsheet, determine if any vasopressor infusions administered. Chart search “bolus”, determine if fluid bolus for hypotension was administered. Open medications list, filter for therapeutic class “autonomic drugs”, determine if administered.
Hypoventilation	Open vitals flowsheet, in “O2 Delivery” row determine if “BIPAP”, “CPAP”, “HFNC”, or “Invasive Ventilation” documented.
ICU admission	Open “ADT Event Report”, determine if patient assigned to a Critical Care service.
Loss of consciousness	Chart search “loss of consciousness” or “syncope”, determine if patient experienced or documented.
Malignant ventricular arrhythmia	Open vitals flowsheet, in “Rhythm” row determine if “VT”, “Vfib”, “Asystole”, or “TdP” is documented. Open any “Code Blue” or “Cardiology consult” notes, determine if malignant ventricular arrythmia is documented or if medications or shocks were administered. Chart search “cardioversion”, determine if administered.
Metallic taste	Chart search “metallic” or “dysgeusia”, determine if patient experienced or documented.
New onset atrial arrhythmia	Open vitals flowsheet, in “Rhythm” row determine if new onset of “PAT”, “SVT”, “AF”, “A-Flut”, or “MAT” is documented.
Perioral numbness	Chart search “perioral numbness” or “numbness”, determine if patient experienced or documented.
Severe/persistent nausea	Chart search “nausea”, determine if at least two notes describe nausea that is not “transient” or at least one episode of nausea is associated with emesis or vomiting.
Seizure	Chart search “seizure” or “convulsing” or “motor activity”, determine if patient experienced or documented. Open any “Neurology consult” notes, determine if seizure or seizure like activity is documented.
Tachycardia requiring treatment	Chart search “cardioversion”, determine if administered for a narrow complex tachycardia. Open medications list, filter for “IV only Amiodarone” or “IV only Beta-adrenergic blocking agents”, determine if administered.
Tinnitus	Chart search “tinnitus” or “ringing”, determine whether patient experienced or documented.

## Results

Primary analysis

**Figure 1:**
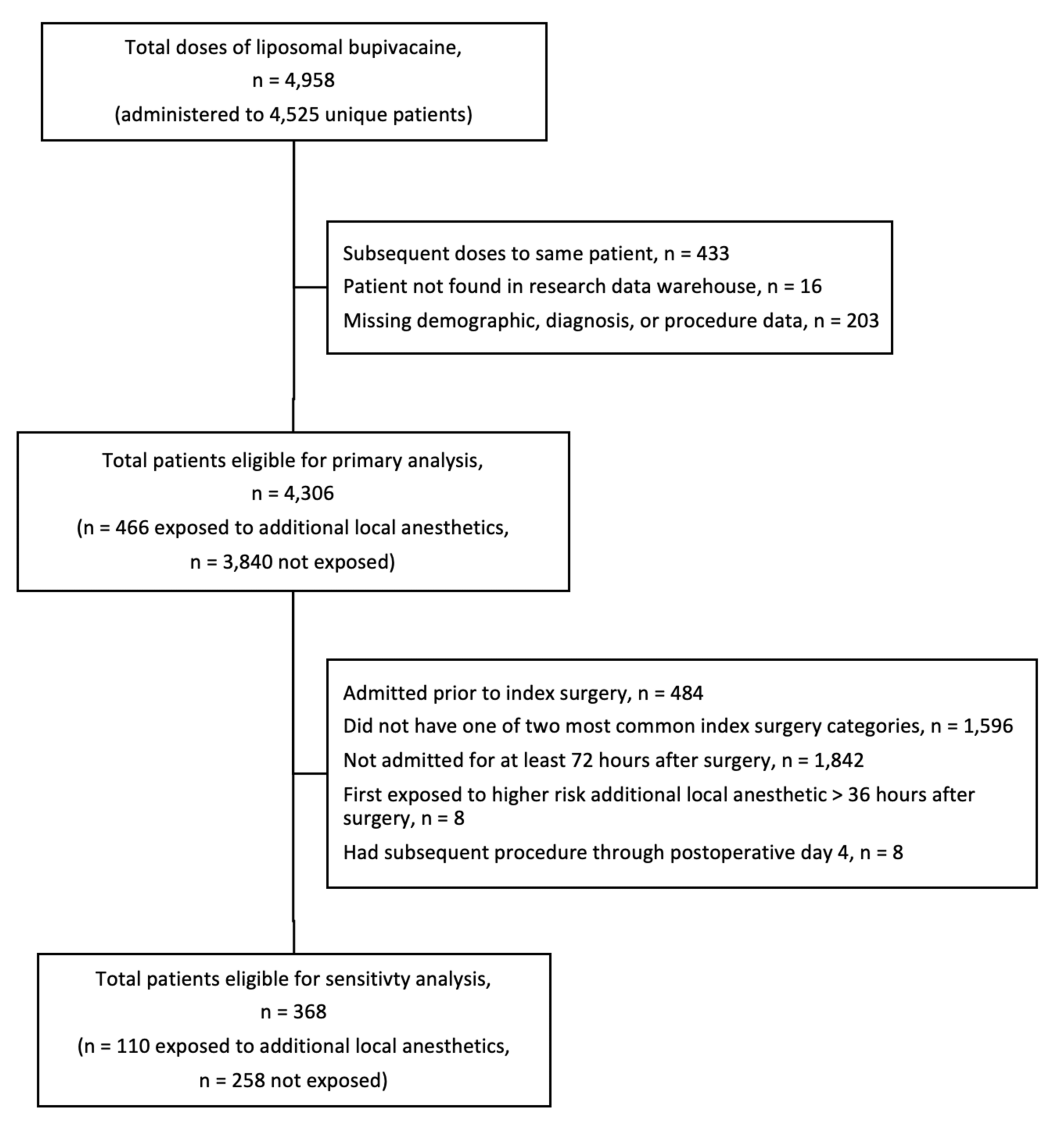
Study flow

We identified 4,958 doses of LB administered to 4,525 unique patients. After excluding patients with incomplete data, 4,306 patients (95.2%) were eligible for inclusion (Figure 1); 466 (12.1% of 4,306) patients received additional local anesthetics within 96 hours after LB administration. Orthopedic surgery was the most common index surgery category for both exposed (260 of 466, 56%) and unexposed (1507 of 3840, 39%) patients (Table 3). The most common exposures were continuous nerve block infusion (157 patients, 33.7% of 466) and intravenous lidocaine injection (96 patients, 20.6% of 466) (Table 4).

**Table 3:** Characteristics of patients exposed and unexposed to additional local anesthetics within 96 hours after LB * Highest ASD for all categories included in “other” ASD: absolute standardized difference, BIPOC: Black, Indigenous, or Person of Color, BMI: body mass index, IQR: interquartile range, LB: liposomal bupivacaine, POD: postoperative day, VW: van Walraven

	Exposed (n = 466)	Unexposed (n = 3840)	Unweighted ASD	Weighted ASD
Patient characteristics				
Median age (IQR) in years	65 (21)	61 (22)	0.16	0.012
Female sex, n (%)	300 (64.4)	2509 (65.3)	0.0096	0.0052
Not English proficient, n (%)	34 (7.3)	335 (8.7)	0.014	0.0019
BIPOC, n (%)	162 (34.8)	1610 (41.9)	0.072	0.0043
Median weight (IQR) in kg	79.9 (27.9)	78.3 (28.4)	<0.001	0.0033
Median BMI (IQR) in kg/m^2^	28.1 (7.2)	27.9 (8.3)	0.16	0.014
Median VW Elixhauser score (IQR)	5 (13)	4 (13)	0.071	0.021
Prior diagnosis of chronic pain, n (%)	134 (28.8)	1377 (35.9)	0.071	0.007
Prior diagnosis of opioid use disorder, n (%)	21 (4.5)	72 (1.9)	0.026	<0.001
Median hours elapsed (IQR), hospital admission to LB dose	5.5 (4.6)	4.7 (4.0)	0.075	<0.001
Median hours elapsed (IQR), LB dose to hospital discharge	74.1 (46.4)	45.8 (49.2)	0.48	0.037
Index surgery category, n (%)				
Orthopedic	260 (55.8)	1507 (39.2)	0.17	0.0036
Gastrointestinal/abdominal	23 (4.9)	608 (15.8)	0.11	0.013
Neurosurgical and orthopedic	61 (13.1)	346 (9.0)	0.041	0.0021
Obstetrics/gynecology	5 (1.1)	244 (6.4)	0.053	0.0072
Skin/breast and hematologic	7 (1.5)	138 (3.6)	0.021	0.0011
Obstetrics/gynecology and hematologic	3 (0.6)	136 (3.5)	0.029	0.0039
Skin/breast	10 (2.1)	126 (3.3)	0.011	0.0011
Urologic	11 (2.4)	99 (2.6)	0.0022	0.0015
Neurosurgical	3 (0.6)	102 (2.7)	0.02	0.0026
Urologic and hematologic	5 (1.1)	66 (1.7)	0.0065	<0.001
Other	78 (16.7)	468 (12.2)	0.014*	0.0043*
Additional procedures, n (%)				
POD 1, major therapeutic	19 (4.1)	69 (1.8)	0.023	0.001
POD 1, major diagnostic/minor therapeutic	5 (1.1)	14 (0.4)	0.0071	<0.001
POD 2, major therapeutic	15 (3.2)	15 (0.4)	0.028	<0.001
POD 2, major diagnostic/minor therapeutic	4 (0.9)	1 (0.03)	0.0083	<0.001
POD 3, major therapeutic	7 (1.5)	19 (0.5)	0.01	<0.001
POD 3, major diagnostic/minor therapeutic	4 (0.9)	3 (0.1)	0.0078	<0.001
POD 4, major therapeutic	4 (0.9)	7 (0.2)	0.0068	<0.001
POD 4, major diagnostic/minor therapeutic	2 (0.4)	2 (0.1)	0.0038	<0.001

**Table 4:** Characteristics of exposures to additional local anesthetics from one to 96 hours after administration of LB Columns may sum to >100% because of patients with multiple exposures. LB: liposomal bupivacaine, IQR: interquartile range

	Exposed patients in primary analysis n = 466	Exposed patients in sensitivity analysis n = 107
Median hours elapsed (IQR), LB dose to first exposure	3.2 (8.7)	2.3 (2.4)
Bolus administrations, patients exposed, n (%)		
Intravenous injection (lidocaine)	96 (20.6)	5 (4.7)
Non-intravenous injection (bupivacaine, mepivacaine, or ropivacaine)	84 (18.0)	9 (8.4)
Non-intravenous injection (LB with/without bupivacaine)	42 (9.0)	5 (4.7)
Non-intravenous lidocaine injection (>5 ml and/or >2% concentration)	27 (5.8)	2 (1.9)
Infusion administrations, patients exposed, n (%)		
Nerve block infusion (bupivacaine, lidocaine, or ropivacaine)	157 (33.7)	59 (55.1)
Subcutaneous/wound infusion (bupivacaine or ropivacaine)	58 (12.4)	19 (17.8)
Intravenous infusion (lidocaine)	55 (11.8)	8 (7.5)
Epidural infusion (bupivacaine or lidocaine)	21 (4.5)	12 (11.2)

**Figure 2:**
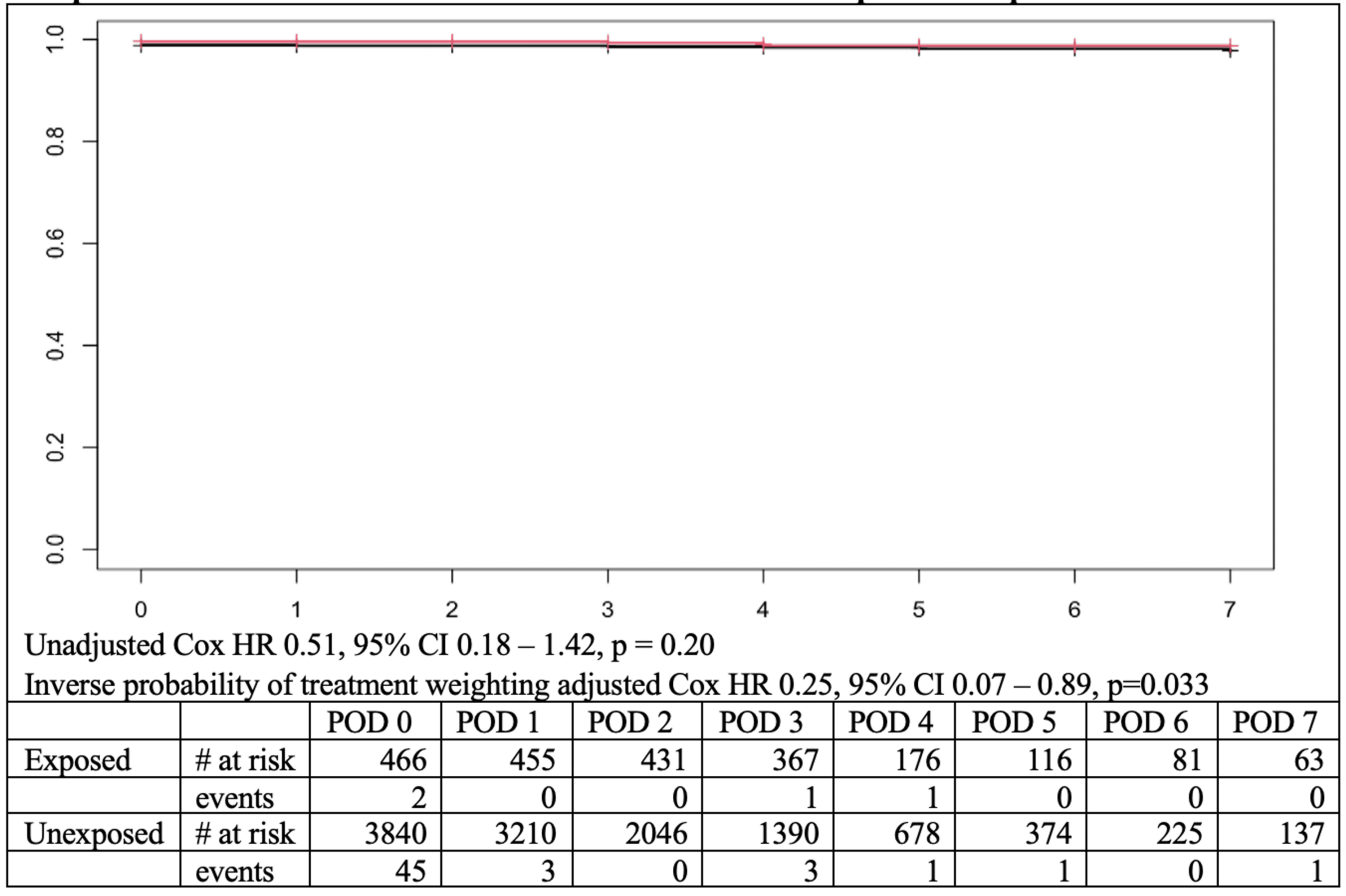
Survival time to possible LAST through POD 7 in patients exposed and unexposed to additional local anesthetics within 96 hours after LB LAST: local anesthetic systemic toxicity, LB: liposomal bupivacaine, POD: postoperative day

There were no instances of formal LAST diagnoses among patients, and no significant difference between exposed and unexposed patients in the cumulative incidence of signs and symptoms of possible LAST (Table 5, 0.9% vs. 1.5%, p = 0.28). The unadjusted survival analysis also showed no significant difference in the hazard of possible LAST between exposed and unexposed patients (Figure 2, HR 0.51, 95% CI 0.18-1.42, p = 0.21).

**Table 5:** Cumulative incidence of possible LAST in patients exposed and unexposed to additional local anesthetics within 96 hours after LB LAST: local anesthetic systemic toxicity, LB: liposomal bupivacaine

	Exposed patients, n = 466	Unexposed patients, n = 3840	p-value
Composite outcome of possible LAST, n (%)	4 (0.9)	57 (1.5)	0.28
Actual LAST diagnosis code (T41.3* or T41.4*)	0	0	n/a
Possible LAST	4 (0.9)	57 (1.5)	0.28
Death	0	4 (0.1)	1
Cardiopulmonary resuscitation (92950)	0	3 (0.08)	1
Lipid administration	0	4 (0.1)	1
Cardiac arrest (I46.9)	0	2 (0.05)	1
Ventricular fibrillation (I49.01) or tachycardia (I47.2)	1 (0.2)	17 (0.4)	0.73
Cardiac complication resulting from procedure (I97.88 or I97.99)	1 (0.2)	7 (0.2)	1
Unspecified convulsion (R56.9)	2 (0.4)	20 (0.5)	1

Exposed patients differed from unexposed patients: they tended to be older, have higher BMIs, have longer lengths of stay after the index surgery, and have their index surgery be more frequently orthopedic and less often gastrointestinal/abdominal. The inverse probability of treatment weighting adequately balanced for these differences, with ASDs consistently <0.05 after weighting (Table 3). The weight-adjusted survival analysis showed a slight decrease in the hazard of possible LAST after exposure (Figure 2; HR 0.25, 95% CI 0.07-0.89, p = 0.033).

Sensitivity analysis

**Figure 3:**
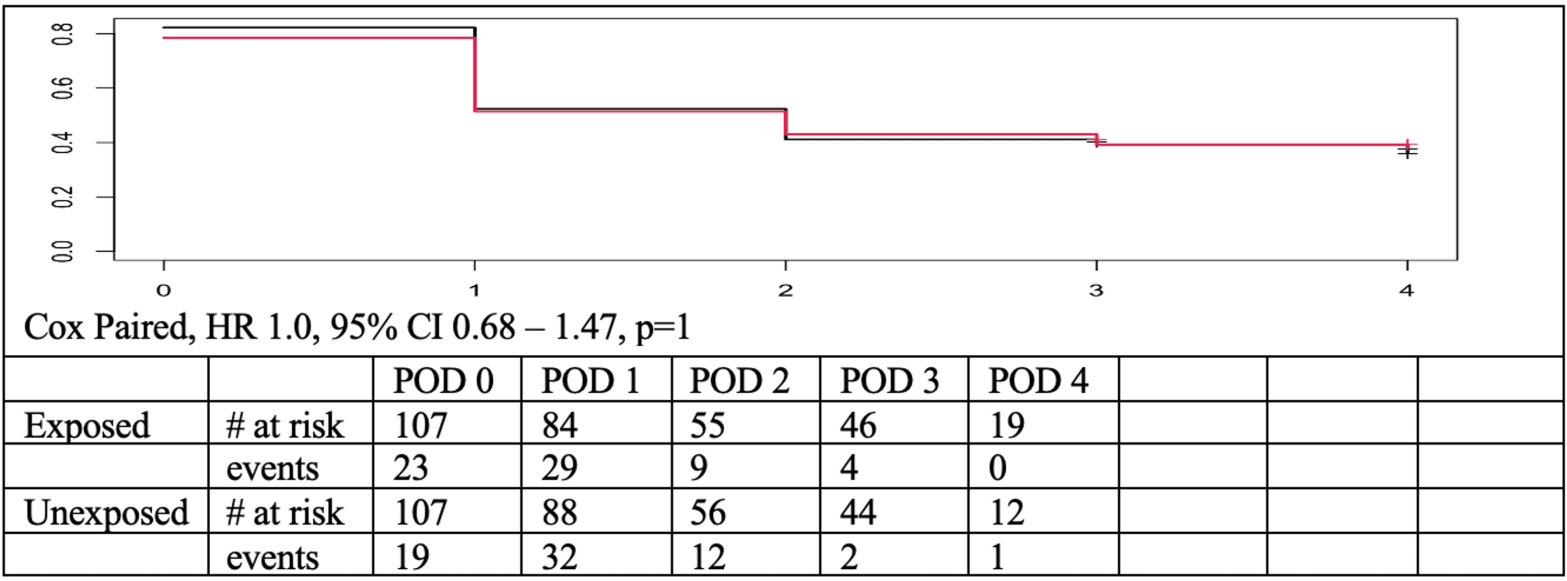
Survival time to possible LAST in the sensitivity analysis LAST: local anesthetic systemic toxicity, POD: postoperative day, HR: hazard ratio, CI: confidence interval

For the sensitivity analysis, from the 4,306 eligible patients in our primary analysis, we excluded 484 patients who were admitted before their index surgery; 1,596 patients who had an index surgery category other than the two most common (orthopedic and gastrointestinal/abdominal); 1,842 patients who were not admitted for at least 72 hours after surgery; eight patients who had subsequent procedures through POD 4; and eight patients who were first exposed to higher risk additional local anesthetic more than 36 hours after surgery. After applying these exclusion criteria, 368 patients remained eligible for inclusion, of which 110 (29.9% of 368) were exposed to additional local anesthetics (Figure 1). After matching on the propensity for exposure, there were 107 (97% of 110) exposed patients in the sensitivity analysis exposed cohort and an equal number in the unexposed cohort, and the cohorts remained well balanced with ASD ≤0.09 (Table 6). There remained no formal diagnoses of LAST, no difference in the cumulative incidence of signs and symptoms of possible LAST (Table 7, p = 1), and no difference in the hazard of possible LAST (Figure 3, HR 1.0, 95% CI 0.68-1.47, p = 1).

**Table 6:** Characteristics of patients included in the sensitivity analysis ASD: absolute standardized difference, SD: standard deviation, BIPOC: Black, Indigenous, and People of Color, BMI: body mass index, VW: van Walraven, IQR: interquartile range, LB: liposomal bupivacaine

	Before matching			After matching		
	Exposed	Unexposed	ASD	Exposed	Unexposed	ASD
Sample size, n	110	258	-	107	107	-
Patient characteristics						
Mean age (SD) in years	63.1 (15.3)	67.6 (13.1)	0.3	64.6 (13.6)	63.9 (14.5)	0.043
Female sex/gender, n (%)	72 (65)	174 (67)	0.042	68 (64)	69 (64)	0.02
Not English proficient, n (%)	10 (9)	26 (10)	0.034	11 (10)	10 (9)	0.033
BIPOC, n (%)	37 (34)	91 (35)	0.035	35 (33)	37 (35)	0.04
Mean weight (SD) in kg	82.3 (20.9)	83.1 (22.7)	0.037	84.4 (22.7)	82.8 (20.9)	0.076
Mean BMI (SD) in kg/m^2^	29.2 (5.8)	29.6 (6.9)	0.058	29.4 (6.8)	29.4 (5.7)	0.011
Mean VW Elixhauser score (SD)	4.8 (7.3)	8.6 (12.0)	0.52	4.9 (9.8)	4.9 (7.4)	0.01
Prior diagnosis of chronic pain, n (%)	28 (25)	118 (46)	0.47	29 (27)	28 (26)	0.022
Prior diagnosis of opioid use disorder, n (%)	7 (6)	8 (3)	0.13	6 (6)	7 (6)	0.034
Median hours elapsed (IQR), LB administration to hospital discharge	98.8 (52.7)	95.6 (47.3)	0.062	96.9 (60.9)	97.5 (52.5)	0.011
Index surgery category, n (%)						
Orthopedic	105 (95)	212 (82)	0.64	104 (97)	102 (95)	0.09
Gastrointestinal/abdominal	5 (5)	46 (18)	0.64	3 (3)	5 (5)	0.09
Additional local anesthetic exposure characteristics						
Median hours elapsed (IQR), LB dose to first exposure	2.3 (2.4)	-	-	2.3 (2.4)	-	-
Exposed to bolus dose, n (%)	10 (9)	-	-	10 (9)	-	-
Exposed to infusion dose, n (%)	94 (85)	-	-	91 (85)	-	-
Exposed to both bolus and infusion, n (%)	6 (6)	-	-	6 (6)	-	-

**Table 7:** Cumulative incidence of possible LAST in the sensitivity analysis LAST: local anesthetic systemic toxicity, ICU: intensive care unit

Exposed/unexposed matched pairs (n = 107)	Occurred in neither member of pair	Occurred in both members of pair	Occurred only in exposed member of pair	Occurred only in unexposed member of pair	p-value
Composite possible LAST	20	44	21	22	1
Serious sign/symptom	101	0	2	4	0.68
Code	104	0	2	1	1
Death	107	0	0	0	n/a
ICU admission	103	0	0	4	0.13
Neurological sign/symptom	21	41	23	22	1
Altered mental status	92	0	7	8	1
Blurry vision	103	0	2	2	1
Dizziness	27	28	24	28	1
Dysarthria	103	0	0	4	0.13
Hypoventilation	103	0	1	3	0.62
Loss of consciousness	103	0	4	0	0.13
Metallic taste	107	0	0	0	n/a
Perioral numbness	107	0	0	0	n/a
Seizure	105	0	2	0	0.48
Severe/persistent nausea	77	5	15	10	0.42
Tinnitus	107	0	0	0	n/a
Cardiac sign/symptom	77	3	10	17	1
Bradycardia requiring treatment	105	0	1	1	1
Hypotension	80	2	9	16	0.23
Malignant ventricular arrhythmia	106	0	0	1	1
New onset atrial arrhythmia	105	0	1	1	1
Tachycardia requiring treatment	109	0	0	1	1

## Discussion

To investigate whether exposure to additional local anesthetics soon after LB is associated with an increased risk of LAST, we used EMR data to examine the medical records of many patients who received LB. We compared the clinical course of patients exposed to additional local anesthetics with that of those not exposed. We identified no cases of LAST among patients who received LB, regardless of whether they were exposed to additional local anesthetics, and no increase in the cumulative incidence of possible LAST among patients exposed to additional local anesthetics compared with those who were not. We anticipated that exposed patients would differ from unexposed patients and used inverse probability of treatment weighting to adjust for patient (e.g., history of chronic pain) and clinical (e.g., postoperative length of stay) factors that could influence whether a patient would be exposed to additional local anesthetics. Our weight-adjusted survival analysis found that patients exposed to additional local anesthetics had a slightly lower hazard of possible LAST. The presentation of LAST can be atypical [23]. We anticipated a high likelihood that clinicians may have missed subtle signs and symptoms of possible LAST. An outcome search strategy relying on administrative coding data would thus underestimate the incidence. This may be particularly true if the treatment team is unaware that a patient received LB hours or days ago and is not on alert for a higher risk of LAST, or if the signs or symptoms of toxicity are mild. Thus, we performed a sensitivity analysis using a manual chart review to identify signs and symptoms of serious neurologic and/or cardiac complications. Similarly, we found no difference in the cumulative incidence of signs and symptoms of possible LAST.

The safety of additional local anesthetic administration soon after LB was called into question at our institution through incident reporting [24], when concerns were raised about subsequent administration of nerve blocks and lidocaine infusions after LB, contrary to FDA labeling. In response, we implemented specific measures to prevent exposure to additional local anesthetics soon after LB, including manufacturer-supplied patient wristbands, head-of-bed signs, and education for physicians and nurses. Additional local anesthetics were inadvertently administered to patients soon after LB, and incidents continued to occur. LB was subsequently removed from our formulary.

LB had been initially added to our hospital formulary in 2014, well before the 2018 labeling change warning against subsequent administration of non-bupivacaine local anesthetics. Hospitals prospectively review new medications for safety before addition to the hospital formulary, but new safety changes to package inserts must be periodically reviewed. Without prospective messaging from the FDA or drug manufacturer, alterations to package inserts may not be flagged for review by hospital formulary and medication safety committees to inform practice changes.

Our study is limited by its single-center retrospective design, the relatively small size of the cohort exposed to additional local anesthetics, and the heterogeneity of our sample, all of which could decrease the generalizability of our findings. Our adjusted survival analysis unexpectedly demonstrated a small but statistically significant decrease in the hazard of LAST in patients exposed to additional local anesthetics. We are unaware of any mechanism by which exposure to additional local anesthetics would reduce the risk of LAST, and this finding may relate to model overfitting or residual confounding.

## Conclusions

In this single-center retrospective cohort study, we used both administrative data and manual chart review to explore the risk associated with administering additional local anesthetics soon after LB. We identified no cases of LAST among patients who received LB, and no increase in the cumulative incidence of possible LAST among patients subsequently exposed to additional local anesthetics compared with those not exposed. Although these findings must be interpreted in light of the relatively small exposed cohort and the inherent limitations of a retrospective, single-institution design, our analysis is reassuring. It suggests that there is unlikely to be a clinically meaningful increase in the hazard of LAST associated with administering additional local anesthetics soon after LB. Larger, multicenter studies and further prospective post-marketing surveillance will be necessary to more definitively characterize this risk and to inform evidence-based formulary and labeling guidance.
